# Cerebroprotective Effect of *Moringa oleifera* against Focal Ischemic Stroke Induced by Middle Cerebral Artery Occlusion

**DOI:** 10.1155/2013/951415

**Published:** 2013-12-03

**Authors:** Woranan Kirisattayakul, Jintanaporn Wattanathorn, Terdthai Tong-Un, Supaporn Muchimapura, Panakaporn Wannanon, Jinatta Jittiwat

**Affiliations:** ^1^Department of Physiology and Graduate School (Neuroscience Program), Faculty of Medicine, Khon Kaen University, Khon Kaen 40002, Thailand; ^2^Integrative Complementary Alternative Medicine Research and Development Center, Khon Kaen University, Khon Kaen 40002, Thailand; ^3^Department of Physiology, Faculty of Medicine, Khon Kaen University, Khon Kaen 40002, Thailand; ^4^Faculty of Medicine, Mahasarakham University, Mahasarakham 44150, Thailand

## Abstract

The protection against ischemic stroke is still required due to the limitation of therapeutic efficacy. Based on the role of oxidative stress in stroke pathophysiology, we determined whether *Moringa oleifera*, a plant possessing potent antioxidant activity, protected against brain damage and oxidative stress in animal model of focal stroke. *M. oleifera* leaves extract at doses of 100, 200 and 400 mg*·*kg^−1^ was orally given to male Wistar rats (300–350 g) once daily at a period of 2 weeks before the occlusion of right middle cerebral artery (Rt.MCAO) and 3 weeks after Rt.MCAO. The determinations of neurological score and temperature sensation were performed every 7 days throughout the study period, while the determinations of brain infarction volume, MDA level, and the activities of SOD, CAT, and GSH-Px were performed 24 hr after Rt.MCAO. The results showed that all doses of extract decreased infarction volume in both cortex and subcortex. The protective effect of medium and low doses of extract in all areas occurred mainly via the decreased oxidative stress. The protective effect of the high dose extract in striatum and hippocampus occurred via the same mechanism, whereas other mechanisms might play a crucial role in cortex. The detailed mechanism required further exploration.

## 1. Introduction

Stroke, the major cause of death and disability, is regarded as the important problem in developing countries [1]. Despite the importance of stroke and the advances of technologies nowadays, clinical therapy of the deliberating disorder is still not in the satisfaction level. Therefore, the prophylactic protection against stroke with neuroprotective agent has gained much attention.

Cerebral ischemia is characterized by a rapid onset of neurological injury due to interruption of blood flow to the brain [2]. This injury has been reported to be associated with the action and interaction of many factors such as excitatory amino acids, calcium overloading, oxidative stress damage, periphery depolarization of infarction, neuroinflammation, and apoptosis [3–5]. However, accumulative lines of evidence in this decade point out to the crucial role of oxidative stress. It has been reported that the reduction of cerebral blood flow and the reperfusion period induce the elevation of oxidative stress and lipid peroxidation [6–9]. Interestingly, both in vitro and in vivo data have demonstrated that this injury can be protected by polyphenolics including flavonoids [7, 8, 10–13].

*Moringa oleifera* Lam. or Marum or Drumstick, a member of Moringaceae family, is widely cultivated in Asia, Polynesia, and the West Indies. In Thailand, leaves of *Moringa oleifera* have been consumed as vegetables for more than 100 years. *M. oleifera* leaves can also serve as a rich source of substance possessing antioxidant activity such as betacarotene, vitamin C, vitamin E, and polyphenolics [14, 15]. Many reports have described the potential therapeutic values of *M. oleifera* including anticancer, antidiabetes, anti-rheumatoid arthritis, anti-fungal, anti-microbial [16], anti-atherosclerotic [17], antifertility, pain relief, depressant [18], diuretic and thyroid regulation effects [19]. Recent findings have shown that the leaves extract also exhibits antioxidant effect and can protect against oxidative damage [20, 21]. In addition, it has been reported that LD_50_ of alcoholic extract of *M. oleifera* leaf is approximately 2.8 g·kg^−1^ BW [22], and this information suggests that it is quite safe even when consumed in a higher quantity due to its high LD_50_. Based on the crucial role of oxidative stress on the pathophysiology of cerebral ischemia and antioxidant effect of *M. oleifera* leaves, the cerebroprotective effect of *M. oleifera* leaves extract against focal ischemic stroke has been focused on. Since no scientific evidence concerning this issue was available, this study was carried out to determine the cerebroprotective effect of the mentioned extract against brain damage and oxidative stress in animal model of focal ischemic stroke.

## 2. Materials and Methods

### 2.1. Plant Material Preparation

The fresh *Moringa oleifera* Lam (Moringaceae) leaves were harvested during November to December, 2010, with the permission from the owners of the land, Mr. Chalerm Pattum, Mr. Padungkiet Jutakanchana, and Mrs. Oranuch Boonlue, in Khon Kaen province, Thailand. The plant specimen was authenticated by Associate Professor Dr. Panee Sirisa-ard, Faculty of Pharmacy, Chiangmai University, Thailand. The voucher specimen was kept at the Integrative Complementary Alternative Medicine Research and Development Center (voucher specimen 2010002), Khon Kaen University, Khon Kaen, Thailand.

### 2.2. Plant Material Preparation

The fresh leaves of *M. oleifera* were immediately cleaned, cut in to small pieces, and dried in oven at 40°C. The dried plant material was ground into powder and extracted with 50% hydroalcohol using maceration technique. Then, the extract was filtered through Whatman filter paper number 1 and evaporated to dryness using rotator evaporator. The yielded extract was kept at 4°C in a dark bottle until used. The percent yield of extract was 17.49%. The extract contained total phenolic compounds at concentration of 86.73–93.6  ±  0.51 mg of GAE·g^−1^ extract. The crude extract was suspended in 1% CMC (sodium carboxymethylcellulose) to the desired concentration during the experiment.

### 2.3. Experimental Animals

Healthy male Wistar rats (300–350 g) were obtained from the National Laboratory Animal Center, Salaya, Nakorn Pathom. They were randomly housed 5 per cage, maintained in 12 : 12 light:dark cycle, and given access to food and water ad libitum. The experiments were strictly performed in accordance with the internationally accepted principles for laboratory use and care of the European Community (EEC directive of 1986; 86/609/EEC). The experiment protocols were approved by the Institutional Animal Care and Unit Committee Khon Kaen University, Thailand (Record no. AEKKU 51/2553). All surgery was performed under the pentobarbital sodium anesthesia in order to minimize animal suffering.

### 2.4. Experimental Protocols

Animals were divided into 7 groups as follows.

 Group I: vehicle plus sham operation group; animals in this group were orally given 1% carboxymethylcellulose and received sham operation.
 Group II: vehicle plus Rt.MCAO group; all rats were orally given 1% carboxymethylcellulose and subjected to the occlusion of right middle cerebral artery.
 Group III: piracetam plus MCAO; rats in this group received Piracetam, a standard drug claiming for the enhanced cerebral blood flow, via oral route at dose of 250 mg·kg^−1^ BW and were exposed to the occlusion of right middle cerebral artery.
 Group IV: vitamin C plus MCAO; the animals in this group received Vitamin C, a well-known antioxidant, via oral route at dose of 250 mg·kg^−1^ BW and were exposed to the occlusion of right middle cerebral artery.
 Group V–VII: *M. oleifera* extract plus MCAO treated groups; rats in these groups were orally given the extract at doses of 100, 200, and 400 mg·kg^−1^ BW and subjected to the occlusion of right middle cerebral artery.

The animals in groups II–VII were orally given the assigned substances at a period of 14 days and subjected to the occlusion of right middle cerebral artery (RT.MCAO), whereas animals in group I were treated with vehicle at the same period and exposed to sham operation. All substances treatments were continually performed throughout a 21-day study period. The assessment of motor and sensory function recovery was performed every 7 days throughout the study period, while the biochemical assays and the determination of histological changes were performed at the end of study.

### 2.5. Focal Cerebral Ischemic Induction

Animals were deprived of food but water was allowed to be assessed 12 hours prior to the surgery. Then, they were anesthetized by injecting thiopental sodium at dose of 60 mg·kg^−1^ body weight via intraperitoneal route. After the anesthetization, the focal cerebral ischemic induction was performed [23]. In brief, the bifurcation of right common carotid artery was exposed through a ventral midline incision. The internal carotid artery and external carotid artery were distally dissected, free from the adjacent tissues, and ligated. The monofilament nylon coated with silicone was gently inserted into the internal carotid artery and the filament was advanced up to 17 mm into the middle cerebral artery (MCA) from carotid bifurcation. Then, the distal end of monofilament was tied up and the wound was sewed using the surgical suture. Rats were cared of until full recovery from anesthesia and returned to cage.

### 2.6. Determination of Neurological Score

The sensorimotor performance of rats following cerebral ischemia induced by right middle cerebral artery occlusion (Rt.MCAO) was evaluated using modified neurological score of Bederson [24] by a “blinded” coworker. Normal rats could extend both forelimbs toward the floor. Rats that extended both forelimbs toward the floor were assigned grade 0 or no spontaneous activity. Cerebral ischemic rats usually flexed the forelimb contralateral to the injured hemisphere. Moreover, it was found that the severely dysfunctional rats had consistently reduced resistance to the gentle lateral push toward the paretic side when the stimulus was applied behind the rats' shoulder. When rats were allowed to move freely, the severely dysfunctional rats usually showed the circling behaviors toward the paretic side. Based on the changes mentioned earlier, the modified neurological score was graded as 6-point neurological function score. In brief, the scoring was performed as follows:

 0: no spontaneous activity,
 1: spontaneous circling,
 2: circling if push tail,
 3: lower resistance to lateral push,
 4: contralateral forelimb flexion,
 5: no apparent deficit.

### 2.7. Hot Plate Test

Hot plate test was used to assess the sensory function recovery of rat by measuring the latency of foot withdrawal reflex in response to temperature stimuli. Rats were placed on the heat surface of hot plate which was maintained at 50°C. Then, the paw withdrawal latency was recorded and used as index indicating the recovery of sensory response to heat stimuli.

### 2.8. Infarction Volume Evaluation

Rats were anesthetized with thiopental sodium at dose of 60 mg/kg BW and transcardially perfused with phosphate buffer solution (PBS). After brain removal, it was coronally cut at 2 mm thick with brain slicer and immersed in 2% 2, 3, 5-triphenyltetrazolium chloride (TTC) solution at room temperature at a 15-minute period. The staining brain sections were photographed, and the infarction area was determined by measuring the white area of brain section with computer software (Image Tool for Window version 3).

### 2.9. Determination of Oxidative Stress Markers

Since the oxidative stresses and free radicals, one of the key factors which induced brain damage in cerebral ischemia, are produced every day via the function of mitochondria [25, 26], polymorphonuclear neutrophils, macrophages, and endothelial cells [27], the alteration of oxidative stress markers has gained much attention. According to the functions of the aforementioned organelle and cells, superoxide was generated and normally it could be detoxified to hydrogen peroxide (H_2_O_2_) by superoxide dismutase (SOD). H_2_O_2_ in turn was converted to water (H_2_O) by catalase (CAT) and glutathione peroxidase (GSH-Px) enzymes. The excess oxidative stress either via the increased production or via the decreased inactivation process of scavenger enzymes could attack the lipid component of cellular membrane and gave rise to the increased lipid peroxidation and neuronal death. Based on the oxidative stress changes mentioned earlier, the lipid peroxidation product such as malondialdehyde (MDA) and the activities of SOD, CAT, and GSH-Px were used as indices to reflect the oxidative stress markers.

To determine the oxidative stress markers, rats were divided into various groups as previously described in the experimental protocol. After the last dose of administration, all rats were sacrificed by the cervical dislocation. Right hippocampus, cortex, and striatum of each rat were isolated and prepared as homogenate for the determination of the oxidative stress markers including malondialdehyde (MDA) level and the activities of the antioxidant enzymes such as superoxide dismutase (SOD), catalase (CAT), and glutathione peroxidase (GPx). MDA level was estimated by determining the accumulation of thiobarbituric acid reactive substances (TBARS) in the brain homogenate [28]. The activities of SOD, CAT, and GSH-Px were determined by recording the ability to inhibit cytochrome C [29], the rate of decrease in H_2_O_2_ [30], and the amount of reduced nicotinamide adenine dinucleotide phosphate (NADPH) oxidized per minute [31], respectively.

### 2.10. Statistical Analysis

Data were presented as mean ± standard error of mean (SEM). Statistical analysis was performed using one-way analysis of variance (ANOVA), followed by LSD posthoc test. Probability levels less than 0.05 were regarded as significant.

## 3. Results

### 3.1. Effect of *M. oleifera* Leaves Extract on the Functional Recovery of Brain Dysfunction

Based on the fact that hemispheric cerebral ischemia due to the occlusion of middle cerebral artery develops contralateral paralysis and sensory loss, the effect of *M. oleifera *leaves extract on the motor and sensory recovery function after Rt.MCAO was determined using neurological examination and hot plate test.

The effect of *M. oleifera *leaves extract on the neurological score had been shown in Table 1. The results showed that rats which received vehicle plus MCAO showed a significant reduction of neurological scores throughout the 21-day experimental period (*P* value < .001 all, compared to vehicle + sham operation). This reduction was reversed by Piracetam throughout the experimental period (*P* value < .05, .001, and .001, resp., compared with vehicle plus MCAO group). Although Vitamin C treated group showed an increased neurological score, the significant effect was observed only at 14 and 21 days after MCAO (*P* value < .01 all, compared with vehicle plus MCAO group). *M. oleifera *leaves extract at doses of 200 and 400 mg/kg BW significantly improved neurological score at the 14 and 21 days after Rt. MCAO, respectively (*P* value < .05 all, compared with vehicle plus MCAO group), whereas *M. oleifera *leaves extract at dose of 100 mg/kg BW treated group failed to produce the significant change.

In addition to the motor performance, the recovery of sensory function after Rt.MCAO was also determined using hot plate test. Data were shown in Table 2. The results showed that vehicle plus MCAO markedly enhanced the foot withdrawal reflex time in hot plate test (*P* value < .001 all, compared to vehicle plus sham operation). Rats exposed to Piracetam plus MCAO showed the significant decreased foot withdrawal reflex time in hot plate test (*P* value < .001, compared to vehicle plus MCAO) at 21-day period after MCAO, whereas Vitamin C treatment showed the significant reduction of this parameter at 7 and 21 days after MCAO (*P* value < .01 and .001, resp., compared to vehicle plus MCAO). It was found that rats which were exposed to the extract at dose of 200 mg/kg BW decreased the elevation of foot withdrawal reflex time induced by MCAO throughout the 21-day period after MCAO (*P* value < .05, .01 and .001, resp., compared to vehicle plus MCAO group), while rats exposed to the extract at doses of 100 and 400 mg·kg^−1^ BW significantly decreased the mentioned parameter at 14 days (*P* value < .05, and .01 resp., compared to vehicle + MCAO) and 21 days after MCAO (*P* value < .01 and .001, compared to vehicle MCAO).

### 3.2. Effect of *M. oleifera* Leaves Extract on Brain Infarction

**Figure 1:**
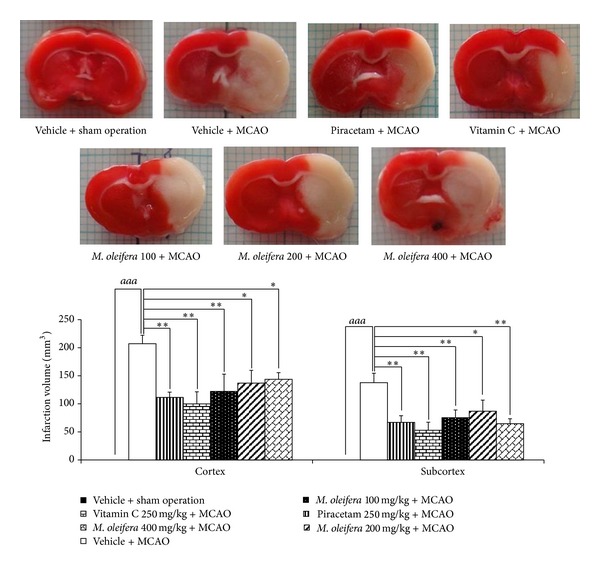
Effect of *Moringa oleifera* leaves extract on brain infarction volume in animal model of focal ischemic stroke. (*n* = 5) Data were expressed as mean ± SEM. ^∗, ∗∗^ *P*-value < .05 and .01 respectively; compared to vehicle plus MCAO.

Figure 1 showed that both rats treated with Piracetam and Vitamin C attenuated brain infarction volume both in cortex and subcortex (*P* value < .01 all, compared to vehicle plus MCAO group). Interestingly, *M. oleifera* leaves extract at doses of 100, 200, and 400 mg·kg^−1^ also decreased brain infarction volume in cortex (*P* value < .01, .05, and .05, resp., compared to vehicle plus MCAO group) and subcortex (*P* value < .01, .05, and .01, resp., compared to vehicle plus MCAO group).

### 3.3. Effect of *M. oleifera* Leaves Extract on Oxidative Stress

**Figure 2:**
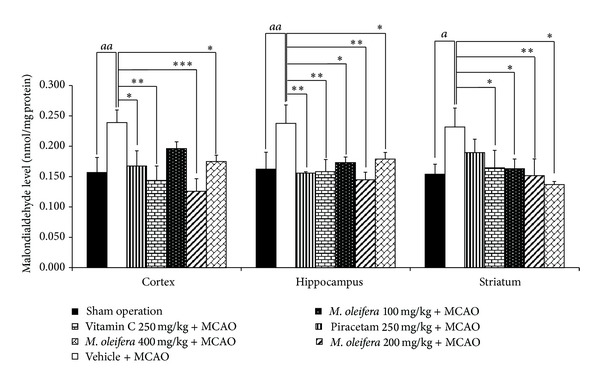
Effect of *Moringa oleifera* leaves extract on malondialdehyde (MDA) level in cerebral cortex, hippocampus and striatum of animal model of focal ischemic stroke. (*n* = 5) Data were expressed as mean ± SEM. ^a, aa^ *P*-value < .05 and .01 respectively; compared to vehicle plus sham operation. ^∗, ∗∗, ∗∗∗^ *P*-value < .05, .01 and .001 respectively; compared to vehicle plus MCAO.

The effect of *M. oleifera* leaves extract on MDA levels in cortex, striatum, and hippocampus was shown in Figure 2. It was found that Rt.MCAO significantly increased the level of MDA in all areas (*P* value < .01, .01, and .05, resp., compared to naïve control group). Piracetam treatment significantly attenuated the elevation of MDA levels induced by MCAO in cortex and hippocampus (*P* value < .05 and .01 resp.; compared to vehicle plus MCAO), whereas Vitamin C treatment significantly alleviated the elevation of MDA levels induced by MCAO in cortex, hippocampus, and striatum (*P* value < .01, .01, and .05, resp., compared to vehicle plus MCAO). *M. oleifera* leaves extract at doses of 200 and 400 mg·kg^−1^BW induced the significant reduction of MDA levels in cortex (*P* value < .001 and .05, resp., compared to vehicle plus MCAO group), hippocampus (*P* value < .01 and .05, resp., compared to vehicle plus MCAO group) and striatum (*P* value < .05 and .01 resp.; compared to vehicle plus MCAO group). However, *M. oleifera* leaves extract at dose of 100 mg·kg^−1^BW significantly attenuated the elevation of MDA levels induced by MCAO only in hippocampus and striatum (*P* value < .05 all, compared to vehicle plus MCAO).

**Figure 3:**
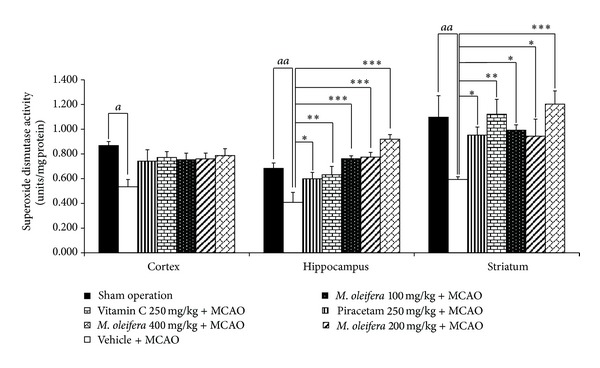
Effect of *Moringa oleifera* leaves extract on superoxide dismutase (SOD) activity in cerebral cortex, hippocampus and striatum of animal model of focal ischemic stroke. (*n* = 5) Data were expressed as mean ± SEM. ^a, aa^ *P*-value < .05 and .01 respectively; compared to vehicle plus sham operation. ^∗, ∗∗, ∗∗∗^ *P*-value < .05, .01 and .001 respectively; compared to vehicle plus MCAO.

In addition to MDA level, the effects of *M. oleifera *leaves extract on endogenous scavenging enzymes mentioned above in ischemic hemisphere were also determined and data were shown in Figure 3. It was found that Piracetam, Vitamin C, and all doses of *M. oleifera *leaves extract treated groups significantly increased the reduction of SOD activity in hippocampus (*P* value < .05, .01, .001, .001, and .001, resp., compared to vehicle plus MCAO) and in striatum (*P* value < .05, .01, .05, .05, and .001, resp., compared to vehicle plus MCAO). Unfortunately, no significant changes of SOD were observed in cortex as shown in Figure 3.

**Figure 4:**
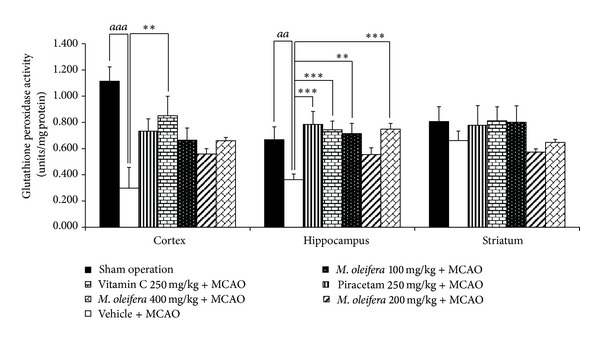
Effect of *Moringa oleifera* leaves extract on glutathione peroxidase (GSH-Px) activity in cerebral cortex, hippocampus and striatum of animal model of focal ischemic stroke. (*n* = 5) Data were expressed as mean ± SEM. ^aa, aaa^ *P*-value < .01 and .001 respectively; compared to vehicle plus sham operation. ^∗, ∗∗, ∗∗∗^ *P*-value < .05, .01 and .001 respectively; compared to vehicle plus MCAO.

Figure 4 showed the effect of *M. oleifera* leaves extract on GSHPx activities in cortex, hippocampus, and striatum. MCAO treated group showed the decreased GSH-Px activity in cortex and hippocampus, but no change was observed in striatum (*P* value < .001 and .01, resp., compared to vehicle plus MCAO). Piracetam treatment significantly attenuated the decreased GSH-Px activity only in hippocampus (*P* value <.001, compared to vehicle plus MCAO group), whereas Vitamin C treatment produced significant attenuation on the reduction of this enzyme in cortex and hippocampus (*P* value < .01 and .001, resp., compared to vehicle plus MCAO). It was found that *M. oleifera* leaves extract at doses of 100 and 400 mg·kg^−1^ BW significantly mitigated the decreased GSH-Px activity induced by MCAO only in hippocampus (*P* value < .01 and .001, resp., compared to vehicle plus MCAO). No changes were observed in other treatments.

**Figure 5:**
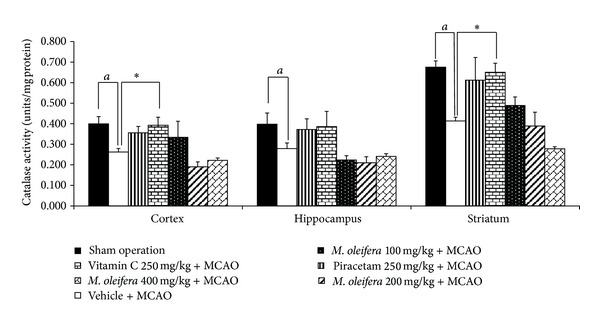
Effect of *Moringa oleifera* leaves extract on catalase (CAT) activity in cerebral cortex, hippocampus and striatum of animal model of focal ischemic stroke. (*n* = 5) Data were expressed as mean ± SEM. ^a, aa^ *P*-value < .05 and .01 respectively; compared to vehicle plus sham operation. *P-value < .05; compared to vehicle plus MCAO.

The effect of *M. oleifera* leaves extract on CAT activity was also investigated and data were shown in Figure 5. MCAO treatment significantly decreased CAT activity in cortex, hippocampus, and striatum (*P* value < .05 all, compared to vehicle treated group). Piracetam treatment failed to show positive modulation effect on CAT activity induced by MCAO, whereas Vitamin C treatment significantly mitigated the decreased CAT activity induced by MCAO in cortex and striatum (*P* value < .05 all, compared to vehicle plus MCAO). However, no significant changes were observed in *M. oleifera* leaves extract treated group.

## 4. Discussion

In this study, we have demonstrated that *M. oleifera* leaves extract attenuated brain dysfunction and brain damage together with the decreased oxidative stress especially in hippocampus and striatum.

It has been well known that oxidative stress is one of the most important factors that exacerbate brain damage induced by cerebral ischemia. Among various brain regions, cortex, striatum, and hippocampus are more susceptible to brain ischemia [32] due to the high rate of oxidative metabolic activity, intense production of reactive oxygen metabolites, high content of polyunsaturated fatty acids, relatively low antioxidant capacity, low repair mechanism activity, and the poor plasticity in the areas just mentioned [33]. Our data clearly demonstrated that the permanent occlusion of MCA produced the decreased SOD and CAT activities in cortex, striatum, and hippocampus, while it produced the significant decreased GSH-Px activity in cortex and hippocampus. These changes in turn decreased the buffering capacity of antioxidant activities and gave rise to the excess oxidative stress and free radicals and resulted in the increased lipid peroxidation indicated by the increased MDA level. Then, the neurodegeneration and brain infarction occurred and finally led to brain dysfunctions of the suffered areas. Vitamin C and Piracetam treatments could protect against brain damage and dysfunctions induced by cerebral ischemia. Previous study demonstrated that Vitamin C could be metabolized to dehydroascorbic acid (DHA) and transported across blood-brain-barrier (BBB) via facilitated transport. DHA could provide the beneficial effect both via its antioxidant effect and its effect to enhance cerebral blood flow [34]. Piracetam was reported to improve mitochondria function and decreased oxidative stress [35]. In addition, Piracetam also enhanced cerebral blood flow [36]. Therefore, both Vitamin C and Piracetam could provide cerebroprotective effect via the antioxidant and the enhanced cerebral blood flow. Our data also demonstrated the decreased oxidative stress by both substances. Therefore, our data are in agreement with the previous data. Both Vitamin C and the plant extract showed a better improvement in foot withdrawal reflex than Piracetam, while Piracetam produced better improvement in neurological score than Vitamin C and the leaves extract Although the precise understanding is still unknown, we did suggest that the recovery of both sensory-motor performance and foot withdrawal reflex might be associated with the different severity of damage and factors which play an important role in brain damage and brain plasticity among various areas. In addition, these factors might also have different vulnerability to the substances such as Vitamin C, the plant extract, and Piracetam. Moreover, the difference in bioavailability of the active form of Vitamin C and Piracetam and active ingredient of the plant extract in the targeted areas might also play an important role in different vulnerability to the effect of the mentioned substance and the different improvement of various brain areas which in turn are possibly to induce different improvement in performance of neurological score and foot withdrawal reflex.

The present study has demonstrated that *M. oleifera* leaves extract treatment at dose of 200 mg·kg^−1^ BW produced optimum changes of MDA level in all areas, whereas the optimum changes of antioxidant enzymes such as SOD and CAT were observed in rats treated with *M. oleifera* leaves extract at dose of 400 mg·kg^−1^ BW. Therefore, our data have shown that no close association between the decreased MDA level and the elevation of antioxidant enzymes was observed. This suggests that *M. oleifera* may decrease oxidative stress especially in cerebral cortex by other mechanisms such as the decreased oxidative stress generation capacity either via mitochondria or via the inflammatory cells [37]. Since *M. oleifera* leaves extract exhibits potent anti-inflammatory activity [38, 39], we suggest that the decreased oxidative stress reflected by the decreased MDA level occurs partly via the anti-inflammatory effect of the plant extract.

It has been found that *M. oleifera* decreases brain infarction volume in cortex without the decreased MDA level. Therefore, our data suggest that other mechanisms also contribute to the role on the neuroprotective effect of *M. oleifera* leaves extract. In addition to oxidative stress, calcium ion over load, excitatory amino acid toxicity, and apoptosis also contribute to the role on neuronal cell death and brain infarction volume [40, 41]. Moreover, the increased blood flow may possibly play a role in the decreased brain infarction volume following stroke [42]. Therefore, the decreased brain infarction volume induced by the extract may be associated with the factors just mentioned.

According to the focal cerebral ischemic stroke model induced by the occlusion of middle cerebral artery occlusion (MCAO), the affected areas are caudate, putamen, parietal cortex, neocortex, and entorhinal cortex [40]. Therefore, the occlusion of MCAO induces both sensory and motor impairments. Since *M. oleifera* leaves extract improved brain infarction both in cortex and striatum, the improvement of both sensory and motor impairments was observed. However, the low dose of extract improved brain infarction volume without the improved neurological score. This suggested that the magnitude of improvement of infarction area was not high enough to improve neurological score. This might be associated with the lack of no significant improvement in striatum.

In this study, no dose dependent response was observed. The possible explanation might be associated with the masking effect of other ingredients in the crude extract of *M. oleifera* leaves. In addition, all parameters investigated in this study appeared to be under the influence of numerous factors. Therefore, no simple relationships between the concentration of extract and the magnitude of changes of the interested parameters were observed.

Although *M. oleifera* leaves extract could not provide better cerebroprotective effect than Vitamin C, it appeared to have low risk of toxicity. It has been shown that *M. oleifera* leaves extract at the dose of more than 3000 mg·kg^−1^ BW is genotoxic, and LD_50_ of alcoholic extract of *M. oleifera* leaves is more than 2800 but less than 5000 mg·kg^−1^ BW [43], whereas the toxicity of Vitamin C was reported at 3000 mg·day^−1^. When compared to Piracetam, *M. oleifera* leaves extract was more easy to approach and cheaper. Therefore, *M. oleifera* leaves extract is an interesting resource for developing functional food and worthy of further study.

## 5. Conclusion

This study has demonstrated that *Moringa oleifera* leaves extract is the potential neuroprotectant which is cheap and easy to approach. The possible underlying mechanism may occur partly via the decreased oxidative stress. Other mechanisms may be also involved and required further exploration.

## Figures and Tables

**Table 1:** Effect of *Moringa oleifera* leaves extract on neurological score in animal model of focal ischemic stroke (n = 5).

Treatment	Neurological score		
	Days after MCAO		
	7 days	14 days	21 days
Vehicle + Sham operation	5.00 ± 0.00	5.00 ± 0.00	5.00 ± 0.00
Vehicle + MCAO	3.33 ± 0.33^aaa^	3.33 ± 0.33^aaa^	3.67 ± 0.33^aaa^
Piracetam + MCAO	4.33 ± 0.33*	4.83 ± 0.17***	5.0 ± 0.0***
Vitamin C + MCAO	3.83 ± 0.40	4.67 ± 0.21**	4.83 ± 0.17**
*M. oleifera *100 mg/kg + MCAO	3.83 ± 0.40	3.67 ± 0.42	4.17 ± 0.40
*M. oleifera* 200 mg/kg + MCAO	4.17 ± 0.31	4.17 ± 0.31*	4.20 ± 0.37
*M. oleifera *400 mg/kg + MCAO	3.83 ± 0.40	4.00 ± 0.45	4.40 ± 0.40*

Data were expressed as mean ± SEM. ^aaa^ *P* value < .001, compared to vehicle plus sham operation. ^∗,∗∗,∗∗∗^ *P* value < .05, .01, and .001, respectively, compared to vehicle plus MCAO.

**Table 2:** Effect of *Moringa oleifera* leaves extract on foot withdrawal time in animal model of focal ischemic stroke (*n* = 5).

Treatment	Foot withdrawal time (seconds)		
	Days after MCAO		
	7 days	14 days	21 days
Vehicle + Sham operation	1.84 ± 0.17	1.70 ± 0.15	1.88 ± 0.16
Vehicle + MCAO	4.30 ± 0.63^aaa^	3.79 ± 0.55^aaa^	3.76 ± 0.20^aaa^
Piracetam + MCAO	3.42 ± 0.35	3.12 ± 0.30	2.73 ± 0.07***
Vitamin C + MCAO	2.48 ± 0.29**	2.98 ± 0.27	2.71 ± 0.08***
*M. oleifera *100 mg/kg + MCAO	3.40 ± 0.27	2.60 ± 0.49*	3.08 ± 0.20**
*M. oleifera* 200 mg/kg + MCAO	3.17 ± 0.24*	2.12 ± 0.33**	2.27 ± 0.11***
*M. oleifera* 400 mg/kg + MCAO	3.43 ± 0.38	2.42 ± 0.28**	2.72 ± 0.24***

Data were expressed as mean ± SEM. ^aaa^ *P* value < .001, compared to vehicle plus sham operation. ^∗,∗∗,∗∗∗^ *P* value < .05, .01, and .001, respectively, compared to vehicle plus MCAO.
